# Body Size Adaptations to Altitudinal Climatic Variation in Neotropical Grasshoppers of the Genus *Sphenarium* (Orthoptera: Pyrgomorphidae)

**DOI:** 10.1371/journal.pone.0145248

**Published:** 2015-12-18

**Authors:** Salomón Sanabria-Urbán, Hojun Song, Ken Oyama, Antonio González-Rodríguez, Martin A. Serrano-Meneses, Raúl Cueva del Castillo

**Affiliations:** 1 Laboratorio de Ecología, UBIPRO, Facultad de Estudios Superiores Iztacala, Universidad Nacional Autónoma de México (UNAM), A.P. 314, Tlalnepantla, 54090 México, México; 2 Department of Entomology, Texas A&M University, 2475 TAMU, College Station, Texas, United States of America; 3 Instituto de Investigaciones en Ecosistemas y Sustentabilidad, UNAM, Morelia, 58190 Michoacán, México; 4 Laboratorio de Biología Evolutiva, Centro Tlaxcala de Biología de la Conducta, Universidad Autónoma de Tlaxcala, Carretera Tlaxcala-Puebla Km. 1.5, 90062, Tlaxcala, México; Scientific Research Centre, Slovenian Academy of Sciences and Arts, SLOVENIA

## Abstract

Altitudinal clines in body size can result from the effects of natural and sexual selection on growth rates and developing times in seasonal environments. Short growing and reproductive seasons constrain the body size that adults can attain and their reproductive success. Little is known about the effects of altitudinal climatic variation on the diversification of Neotropical insects. In central Mexico, in addition to altitude, highly heterogeneous topography generates diverse climates that can occur even at the same latitude. Altitudinal variation and heterogeneous topography open an opportunity to test the relative impact of climatic variation on body size adaptations. In this study, we investigated the relationship between altitudinal climatic variation and body size, and the divergence rates of sexual size dimorphism (SSD) in Neotropical grasshoppers of the genus *Sphenarium* using a phylogenetic comparative approach. In order to distinguish the relative impact of natural and sexual selection on the diversification of the group, we also tracked the altitudinal distribution of the species and trends of both body size and SSD on the phylogeny of *Sphenarium*. The correlative evidence suggests no relationship between altitude and body size. However, larger species were associated with places having a warmer winter season in which the temporal window for development and reproduction can be longer. Nonetheless, the largest species were also associated with highly seasonal environments. Moreover, large body size and high levels of SSD have evolved independently several times throughout the history of the group and male body size has experienced a greater evolutionary divergence than females. These lines of evidence suggest that natural selection, associated with seasonality and sexual selection, on maturation time and body size could have enhanced the diversification of this insect group.

## Introduction

Body size relates to many aspects of an organism’s biology, such as local adaptations to different climatic conditions, female fecundity and male mating success [1]. Local adaptations to different climatic conditions (e.g. temperature and season length) affect body size through the regulation of growth rates and development times [2,3]. Growth rates are positively affected by temperature, food quantity and quality [4]; whereas development time is largely constrained by seasonality, which determines the duration of optimal developmental conditions, such as temperature and food availability [5,6]. The regulation of growth rates and development times are under strong natural selection due to seasonality. Generally, seasonality increases with elevation, constraining the available time for development and reproduction [7,8]. This can favor the evolution of shorter development times, decreased time to reach maturity, and smaller adult body size at high elevations than in lowlands [7–10]. Nonetheless, the fitness benefits of decreasing the time to reach maturity may be counterbalanced by the costs of small size on the reproductive success of females and males [10–12].

Female fecundity and male mating success usually increase with body size [13–15]. However, the size that conveys maximal fitness (i.e. the optimal body size) often differs between the sexes, thus generating sexual size dimorphism (SSD). Male-biased SSD can result when male mating success increases with size due to male–male competition or female choice [16], or due to allocation to reproductive reserves, such as nuptial gifts or ejaculate size [17]. Conversely, female-biased SSD results when large females have higher fecundity [15], or small males have advantages in mate searching or courting due to a higher agility [18–20]. In most taxa examined for these relationships, strong correlations between the sexes have been found (typically > 0.9) [16,21], which are likely to arise because of high genetic correlations between males and females [22]. Despite these high correlations, the magnitude of SSD often varies considerably among closely related species, indicating some independence in the evolutionary trajectories of body size between the sexes [23].

In many taxa, the magnitude of SSD changes systematically with mean body size, either increasing or decreasing as body size increases [21,24,25]. The former pattern is common in taxa where males are larger than females, while the latter occurs in some species in which females are the larger sex. Both patterns are explained by greater evolutionary divergence in male size, compared with female size; such pattern is known as Rensch’s rule [23,24]. For many taxa this allometric trend can be attributed to sexual selection acting on male body size [3,21,26]. The converse trend, where female size varies more than male size, is less common, but seems to be the result of strong fecundity selection acting on females [25,27,28].

Despite the evolutionary implications of natural selection and sexual selection on population differentiation and speciation, few studies have explored the relative impact of altitudinal climatic variation on the diversification of Neotropical insects [7,8,29–31]. Regions with topographical heterogeneity provide opportunities to study the adaptive value of body size in response to climatic variables [8,31,32]. In these regions, seasonality, temperature, and precipitation regimes can vary considerably with altitude, allowing the settlement of different climates in short distances [8,33]. This makes local adaptations possible only if selection is strong enough to neutralize the expected continual gene flow from adjacent populations [32]. Most comparative studies addressing the interspecific variation of body size and SSD in relation to altitudinal climatic gradients have not considered the phylogenetic relationships among species [34], which allows separating the effects of a common evolutionary history from the relative impact of natural and sexual selection [35].

In this study, we investigate the relationship between altitudinal climatic variation and body size, and the divergence rates of body size between females and males in Neotropical grasshoppers of the genus *Sphenarium* (Orthoptera: Pyrgomorphidae), taking into account their phylogenetic relationships. Because the season length limits the body size grasshoppers can achieve, we would expect large adult sizes at lower elevations associated with high temperature and precipitation regimens. In addition, if sexual selection acting on male body size has been stronger than fecundity selection acting on females, a greater evolutionary divergence in male size than in female size would be expected.

## Methods

### Study species

The genus *Sphenarium* Charpentier, 1842 is distributed from central Mexico to northwestern Guatemala and represents the most diverse group of the American Pyrgomorphidae [36,37]. Currently, eight taxa are recognized within this genus: *S*. *mexicanum mexicanum*, *S*. *mexicanum histrio*, *S*. *purpurascens purpurascens*, *S*. *purpurascens minimum*, *S*. *borrei*, *S*. *macrophallicum*, *S*. *rugosum* and *S*. *variabile* [38]. These species are flightless, polyphagous, and univoltine [37]. Their nymphs emerge principally in the beginning of the rainy season (around mid-May) and adults die in the winter (from mid-December to mid-February) [39–42].

*Sphenarium* grasshoppers represent a good model system to explore the relative impact of altitudinal climatic variation on body size adaptations. These grasshoppers have a wide altitudinal distribution, ranging from the sea level to approximately 2600 m, across the climatically heterogeneous Mexican topography, with extensive inter- and intraspecific body size variation [36,37,43]. Moreover, males and females of this genus are highly dimorphic (e. g. the thorax is wider in females and forelegs are wider in males). In addition, in *S*. *purpurascens*, maturation time and body size are under strong natural and sexual selection [44,45], and there is a positive relationship between body size and fecundity [46].

### Ethical statement

In Mexico grasshoppers specimens were collected under the permit SGPA/DGVS/032887/13 issued by Secretaría de Medio Ambiente y Recursos Naturales; Dirección General de Vida Silvestre. Samples from Guatemala were provided by the Universidad del Valle de Guatemala Collection of Arthropods and exported through the permit granted to Enio Cano by Consejo Nacional de Áreas Protegidas. The Dominican Republic government through Ministerio del Medio Ambiente y Recursos Naturales provided the necessary permits for collecting and exportation in Jaragua National Park to Hojun Song. The field studies did not involved endangered or protected species.

### Fieldwork

Between 2008 and 2013 we collected *Sphenarium* grasshoppers from 63 localities across their geographic distribution throughout central and southern Mexico. Collection sites varied in elevation from 15 to 2571 m above the sea level (m.a.s.l.) (See S1 Table). Geographic position and elevation of each locality was recorded during fieldwork with a GPS-map 60CSx (Garmin, Kansas City, USA). All collected specimens were stored at -80°C in individual vials and vouchered (See S1 Table) in the Laboratory of Genetic and Molecular Ecology, IIES [Universidad Nacional Autónoma de México (UNAM), Morelia] and Laboratory of Ecology, UBIPRO (FES-Iztacala; UNAM). Specimens are available upon request to the corresponding author.

In this study we included the eight recognized taxa of *Sphenarium*, as well as the two intermediate forms (between *S*. *p*. *purpurascens* and *S*. *p*. *minimum*; and between *S*. *m*. *mexicanum* and *S*. *m*. *histrio*) identified by Boyle [36] and Kevan [37]. Our taxonomic identifications were based on the most recent taxonomic work for the genus [36,37] and by comparing our samples with identified museum specimens and types housed at UNAM’s Collection of Insects (Mexico City), the University of Michigan Museum of Zoology (Ann Arbor, USA), and the Academy of Natural Sciences of Drexel University (Philadelphia, USA). S1 Table further provides information on the taxonomic identification of collected specimens.

### Acquisition of genetic information

We extracted genomic DNA from single hind femur of one to three specimens from each sampled locality using Qiagen DNeasy kit (Qiagen, Valencia, USA). We amplified fragments of three mitochondrial loci [*Cytochrome c Oxidase subunit 1* (CO1) and *subunit 2* (CO2), and the *12 Subunit of ribosomal RNA* (12S)] and two nuclear loci [*Histone 3* (H3) and the *Internal Transcribed Spacer between 5*.*8S rRNA and 28S rRNA* (ITS2)]. For mitochondrial loci, we followed the recommendations of Song et al. [47] to avoid co-amplification of nuclear mitochondrial pseudogenes. Two long fragments of 3600 (F1) and 2100 (F2) base pairs (bp) of the mitochondrial genome were amplified and used as templates for nested-PCR amplifications of mitochondrial targets (F1 for CO1 and CO2; and F2 for 12S). We provide information about the primers used on Table 1. All PCR reactions were performed using Elongase Enzyme mix (Invitrogen Corporation, Carlsbad, USA) following the manufacturer’s recommendations. For nested-PCRs we used as a template: 1:10 dilution for each Long-PCR product (F1 and F2). Long-PCR conditions included 2 min of initial denaturation at 92°C; 39 cycles of 92°C for 30 s, 50°C for 30 s, 60°C for 5 min; and a final extension at 60°C for 20 min. Other PCR reactions were performed using an initial denaturation at 92°C for 2 min, followed by 34 cycles of 92°C for 30 s, 30 s at the specific Tm for each primer combination (see Table 1), 2 min at 60°C; and a final extension at 60°C for 10 min. Single-band PCR products were purified using PrepEase Purification 96-well plate kit (USB Corporation, Santa Clara, USA) and samples containing more than a single band were purified using QIAquick Gel Extraction Kit (Qiagen, Valencia, USA).

**Table 1:** List of amplified loci indicating their approximate size (bp), annealing temperature (Tm) and the pairs of primers used for the PCR reactions.

Loci	Size (bp)	Tm (°C)	Primer ^A^	Sequence (5'-3')
F1	3600	50	ORMET [48]	CATAAGCTAATGGGTTCATAC
			ORRLYS [48]	GAGACCAGTACTTGCTTTCAGTCATC
F2	2100	50	OR16SN ^B^	AGAAACCGACCTGGCTCACGC CGG
			OR12SN ^B^	CGTGCCAGCAGCCGCGGTTATACG
CO1	1180	58	SPHCO1F ^C^	TAGATCATCAATGGTTAATACAGG
			SPHCO1R ^C^	CTGATATGAGTGTTCTGCAGGAGG
CO2	550	58	C2J3138 [49]	GGAGCTTCACCATTAATAGAACA
			C2N3661 [49]	CCACAAATTTCTGAACATTGACCA
12S	360	58	SRJ14233 [49]	AAGAGCGACGGGCGATGTGT
			SRN14588 [49]	AAACTAGGATTAGATACCCTATTAT
H3	329	60	HexAF [50]	ATGGCTCGTACCAAGCAGACGGC
			HexAR [50]	ATATCCTTGGGCATGATGGTGAC
ITS2	320	60	CAS5p8sFc [51]	TGAACATCGACATTTYGAACGCACAT
			CAS28sB1d [51]	TTCTTTTCCTCCSCTTAYTRATATGCTTAA

^A^ source of primers is indicated within brackets and superscript letters.

^B^ designed by H. Song

^C^ designed by S. Sanabria-Urbán

We sequenced both strands of each purified products using BigDye Terminator v3.1 Cycle Sequencing Kit (Applied Biosystems, Foster City, CA, USA) using Applied Biosystems 3730XL DNA Analyzer (Applied Biosystems, Foster City, USA). We analyzed forward and reverse sequences of each sample with SEQUENCHER v. 4.2 (Gene Codes Corporation, Ann Arbor, USA) and aligned them in MUSCLE [52], using default parameters. We translated and checked for stop-codons the coding loci sequences (CO1, CO2 and H3) using MEGA v. 6.0.6 [53]. All sequences were deposited on GenBank (see S1 Table for accession numbers).

In addition, we incorporated genetic information from the closest relatives of *Sphenarium* in America (including *Prosphena scudderi* and *Jaragua oviedensis*) and Asia (including *Mekongiana xiangchengensis*, *Mekongiella kingdoni*, *M*. *xizangensis* and *Yunnanites coriacea*) [37,54], as well as other American Pyrgomorphidae (*Pyrgotettix pueblensis*, *Sphenotettix nobilis*, *Sphenacris crassicornis*) and Acridoidea (*Schistocerca gregaria gregaria*). Genetic information of these outgroup taxa were primarily obtained for the present study or by retrieving the available information from the GenBank (See S1 Table for outgroup species information).

We constructed a dataset comprising the total genetic information obtained from 67 ingroup and 15 outgroup individuals. We subdivided this dataset in 11 partitions corresponding to the 1^st^, 2^nd^ and 3^rd^ codon positions of each coding loci (CO1, CO2 and H3) and the two non-coding loci (12S and ITS2). We estimated the best partitioning scheme for this dataset and models of nucleotide substitution for each partition using the greedy algorithm implemented in PARTITIONFINDER v. 1.1.1 [55]. The final dataset was subdivided in seven partitions (P1-P7) corresponding to the 1^st^ codon position of CO1 (P1), the 2^nd^ codon position of CO1 and CO2 (P2), the 3^rd^ codon position of CO1 and CO2 (P3), the 1^st^ codon position of CO2 and the 12S locus (P4), the 1^st^ and 2^nd^ codon position of H3 (P5), the 3^rd^ codon position of H3 (P6), and the ITS2 locus alone (P7). We individually applied five substitution models to each partition: JC+I for P5, HKY+G for P6 and P7, HKY+I+G for P2, GTR+G for P1 and P3 and GTR+I+G for P4.

### Phylogenetic reconstruction

We conducted a concatenated Bayesian inference (BI) analysis in MRBAYES v. 3.2.6 [56] with the total genetic evidence dataset obtained, applying the specific substitution model estimated for each partition. This analysis consisted of four independent runs, each of them with 10,000,000 generations and four chains, sampling each 1000 generations. We used default priors for other parameters in the analysis. We assessed parameter convergence and proper mixing of independent runs using TRACER v.1.6 [57]. We also discarded 25% of the samples obtained prior to stability as burn-in.

**Fig 1:**
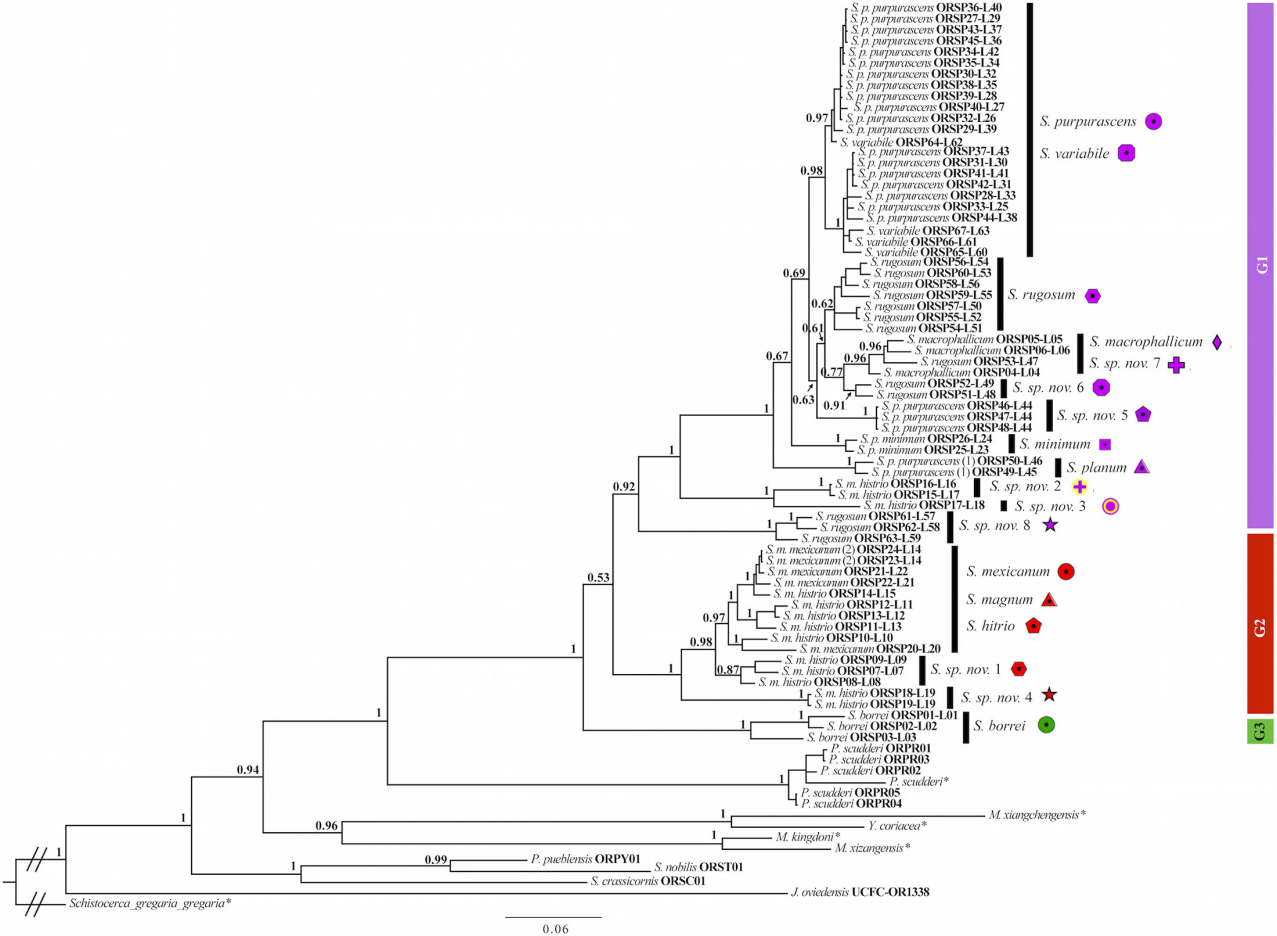
Phylogeny based on a concatenated Bayesian analysis of the total genetic evidence retrieved from 67 *Sphenarium* and 15 outgroup taxa. Tip labels indicate current taxonomic classification, voucher numbers and locality ID for all included terminals, except for those whose genetic information was retrieved from GenBank (*). Black vertical bars indicate the phylogenetic position of the identified species based on our integrative taxonomy approach (names and coloured symbols in front of the black bars).

This initial phylogenetic analysis (Fig 1) indicated that current taxonomic classification [38] of *Sphenarium* species did not reflect their evolutionary relationships. Most currently recognized species were paraphyletic, and only *S*. *borrei* and *S*. *p*. *minimum* were recovered as monophyletic taxa. In addition, we identified three broad patterns of divergence in *Sphenarium*. The first pattern was that some taxa were well-defined by male genital morphology, despite the fact that they were genetically close. The second pattern was that our molecular data revealed cryptic diversity among genetic lineages that were morphologically similar. Finally, the third pattern was that some taxa were both morphologically and genetically differentiated.

In order to define the taxonomic units for our comparative analysis, we adopted an integrative taxonomy framework in identifying lineages that would potentially represent valid species within *Sphenarium*. Specifically, we recognized as species those taxa that could be consistently identified on the basis of their male genital morphology, as noted previously by Boyle [36] and Kevan [37]. We followed this criterion because the male genitalia morphology is widely used in defining species concepts in grasshoppers [58]. Moreover, these morphological species shared no mitochondrial haplotype in our genetic dataset despite they were genetically close. We also recognized as different species those specimens that formed well-supported monophyletic groups (posterior probability values higher than 0.85) and/or divergent lineages that were geographically structured in the concatenated phylogenetic reconstruction (Fig 1). These identified genetic species also diverged from other species with *P-distance* values greater than 2% in pairwise comparisons of their *CO1* sequences (See S2 Table), which is concordant with the *CO1* interspecific levels of differentiation observed in other invertebrates [59]. Finally, we considered as species those taxa that were both morphologically and genetically differentiated. After we identified the lineages that could represent valid species within *Sphenarium* using the integrative taxonomy framework (see Results section for details), we used this taxonomic classification for further analyses.

Particularly, the black vertical bars indicating the phylogenetic position of *S*. *purpurascens*, *S*. *variabile*, *S*. *macrophallicum*, *S*. *sp*. *nov*. *7*, *S*. *mexicanum*, *S*. *magnum* and *S*. *histrio* represent cases where species differentiation was primarily morphological and they did not separate in individual monophyletic groups (See methods and results sections for details). The numbers positioned closely to the nodes indicate posterior probability values. G1, Monophyletic Group 1; G2, Monophyletic Group 2; G3, Monophyletic Group 3. *S*. *p*. *purpurascens* (1) intermediate form between *S*. *p*. *purpurascens* and *S*. *p*. *minimum*. *S*. *m*. *mexicanum* (2) intermediate form between *S*. *m*. *mexicanum* and *S*. *m*. *histrio*.

We also estimated a species tree of the *Sphenarium* taxa identified using the multilocus coalescent-based Bayesian approach implemented in *BEAST v. 1.8.1 [60]. This species tree approach incorporates uncertainty associated with gene trees due to incomplete lineage shorting, nucleotide substitution model parameters and coalescent process [61]. For this analysis, we used a smaller dataset comprising all nucleotide sequences of *Sphenarium* (67 individuals) and *P*. *scudderi* (6 individuals), which were recovered as sister taxa in our previous concatenated phylogenetic reconstruction. We used the same partitioning scheme and nucleotide substitution models previously specified. We applied an uncorrelated relaxed clock with lognormal distribution and set a Birth Death Model as the tree prior, using a Piecewise linear and constant root for population size prior. We set the length of the Markov chain Monte Carlo at 100 million generations, sampling every 10,000 generations and two independent runs. We verified parameter convergence and proper mixing of the independent runs using TRACER 1 v 1.6 [57]. We constructed a consensus species tree setting a burn-in at 25%.

### Estimation of climatic parameters

Adult body size is affected by seasonality, temperature, and food availability. Food availability for primary consumers in food webs relies on plant primary production, which strongly depends on precipitation regimens [62,63]. For this reason, we considered the mean regional temperature and precipitation parameters associated with each collecting site. We obtained Mean Temperatures of the Wettest (MTWT) and the Coldest Trimesters (MTCT); and Mean Annual Precipitation (MAP) and Temperature Seasonality (TS) values from high-resolution monthly climate surfaces for Mexico [64] (Table 2). TS represents the amount of temperature variation over a year based on the ratio of the standard deviation of the monthly mean temperatures to the mean monthly temperature (also known as the coefficient of variation, CV). Thus, the larger the TS values the greater the variability of the temperature [65]. Similar climatic parameters have been used in other studies on body size variation in altitudinal clines [e.g. 34]. On the other hand, we used MTWT and MTCT since they encompass approximately the temperature regimens associated with *Sphenarium* life cycles, from the beginning of the rainy season (MTWT) to the beginning of the winter (MTCT). For each identified species, we estimated mean values of elevation and climatic parameters considering the collecting point information of each individual within the taxa (Table 2). All values, except for TS, were log-transformed for subsequent analysis.

**Table 2:** Mean values of elevation and climatic parameters of the identified *Sphenarium* species.

Species	*N*	NL	Elevation (m.a.s.l.)	TS (CV)	MTWT (°C)	MTCT (°C)	MAP (mm)
*S*. *borrei*	50	3	1477.24	0.82	21.67	16.44	950.57
*S*. *histrio*	91	5	671.32	0.50	23.76	20.98	1011.40
*S*. *macrophallicum*	60	3	953.66	0.65	24.17	21.56	1047.60
*S*. *magnum*	58	1	68.13	0.50	28.40	25.55	878.23
*S*. *mexicanum*	48	3	134.75	0.73	26.35	22.30	1901.24
*S*. *minimum*	33	2	1458.96	0.68	18.92	14.73	1725.30
*S*. *planum*	24	2	1760.83	0.69	19.75	15.15	476.74
*S*. *purpurascens*	3967	19	2147.70	0.72	17.79	13.36	749.93
*S*. *rugosum*	790	8	1635.25	0.54	20.01	17.49	1017.38
*S*. *variabile*	63	4	1539.10	0.48	19.42	16.90	697.75
*S*. *sp*. *nov*. 1	59	3	111.25	0.49	27.99	25.34	956.02
*S*. *sp*. *nov*. 2	62	2	1073.74	0.46	23.41	21.22	1338.79
*S*. *sp*. *nov*. 3	17	1	216.00	0.40	26.32	24.80	1421.49
*S*. *sp*. *nov*. 4	27	1	1145.00	0.83	22.14	16.88	1356.36
*S*. *sp*. *nov*. 5	7	1	1336.00	0.53	21.44	18.93	1189.35
*S*. *sp*. *nov*. 6	23	2	1619.82	0.59	20.48	17.74	955.05
*S*. *sp*. *nov*. 7	20	1	730.00	0.53	24.84	23.37	1141.21
*S*. *sp*. *nov*. 8	42	3	621.30	0.60	25.88	22.87	987.43

n, number of individuals considered; NL, number of localities considered; TS, Temperature Seasonality; MTWT, Mean Temperature of The Wettest Trimester; MTCT, Mean Temperature of The Coldest Trimester; MAP, Mean Annual Precipitation; CV, Coefficient of Variation.

### Morphological measurements

Using a digital calliper (Mitutoyo Corp., Tokyo, Japan), we measured Femur I Width, Femur III Length, and Thorax Length and Width of each collected adult male and female of *Sphenarium* from the 63 sampled localities. These traits are known to be under natural and sexual selection in *S*. *purpurascens* [44,45]. In this study we assumed that body size was positively correlated with maturation time in all species. This assumption is true for at least two species in the genus, *S*. *purpurascens* and *S*. *histrio* (Cueva del Castillo, *Obs*. *Pers*.). Considering the total number of individuals for each taxon, we averaged the values of the four morphological traits per species and sex (Table 3). These values were then log-transformed before they were used in the comparative analyses. In *Sphenarium* species Femur I is larger in males than females, whereas thorax width and length are larger in females than males. However, Femur III shows a mixed SSD pattern (see below). Due to this interspecific variation in morphological traits, we used the Lovich and Gibbons Sexual Dimorphism Index (SDI) [66] to estimate the magnitude and direction of SSD in *Sphenarium*. We obtained the SDI for each morphological trait and species (Table 3) dividing females’ (the larger) on males’ (the shorter) mean trait values, and then subtracting 1. Thus, positive values indicate female biased SSD and negative values indicate male biased SSD (Table 3).

**Table 3:** Mean values of body size measurements and Sexual Dimorphism Index (SDI) of identified *Sphenarium* species. Numbers in bold and underlined indicate maximum and minimum mean values observed for each trait measured, respectively.

	*n*		Females (mm)				Males (mm)				Species (mm)				Sexual dimorphism index (♀/♂)			
**Species**	**♀**	**♂**	FIW	FIIIL	TL	TW	FIW	FIIIL	TL	TW	FIW	FIIIL	TL	TW	FIW	FIIIL	TL	TW
*S*. *borrei*	24	26	1.07	13.64	7.21	9.56	1.28	12.94	5.75	6.74	1.18	13.27	6.45	8.09	-0.16	0.05	0.25	0.42
*S*. *histrio*	46	45	1.05	14.19	7.1	9.1	1.31	12.97	5.74	6.39	1.17	13.59	6.43	7.76	-0.20	0.09	0.24	0.42
*S*. *macrophallicum*	30	30	1.19	15.27	8	9.96	1.53	14.67	7.2	7.96	1.36	14.97	7.6	8.96	-0.22	0.04	0.11	0.25
*S*. *magnum*	32	26	1.18	15.99	7.33	9.51	1.62	15.7	6.77	7.69	1.39	15.86	7.07	8.67	-0.27	0.02	0.08	0.24
*S*. *mexicanum*	24	24	**1.38**	**18.51**	**9**	**11.42**	**1.76**	**16.73**	**7.59**	8.42	**1.57**	**17.62**	**8.29**	9.92	-0.22	0.11	0.19	0.36
*S*. *minimum*	16	17	0.99	12.85	6.62	8.82	1.17	11.45	4.96	5.77	1.08	12.13	5.76	7.25	-0.15	0.12	0.33	0.53
*S*. *planum*	12	12	0.96	12.33	6.66	9.08	1.11	10.69	4.77	5.87	1.03	11.51	5.71	7.48	-0.14	**0.15**	**0.40**	**0.55**
*S*. *purpurascens*	1797	2170	0.85	12.07	6.02	7.94	1.3	12.62	5.54	6.64	1.09	12.37	5.76	7.23	**-0.35**	-0.04	0.09	0.20
*S*. *rugosum*	378	412	1.08	14.62	7.06	9.04	1.44	14.38	6.5	7.64	1.27	14.49	6.77	8.31	-0.25	0.02	0.09	0.18
*S*. *variabile*	29	34	0.91	11.82	5.9	8.8	1.17	10.95	4.74	5.99	1.05	11.35	5.27	7.28	-0.22	0.08	0.24	0.47
*S*. *sp*. *nov*. 1	27	32	1.29	17.15	8.6	10.56	1.62	15.67	7.18	8.13	1.47	16.35	7.83	9.24	-0.20	0.09	0.20	0.30
*S*. *sp*. *nov*. 2	29	33	1.14	14.59	7.78	9.98	1.45	14.05	6.47	7.68	1.31	14.3	7.08	8.76	-0.21	0.04	0.20	0.30
*S*. *sp*. *nov*. 3	6	11	1.02	13.8	6.93	8.89	1.27	12.76	5.8	6.51	1.18	13.13	6.2	7.35	-0.20	0.08	0.19	0.37
*S*. *sp*. *nov*. 4	10	17	1.25	15.22	8.06	10.98	1.57	14.45	6.85	8.42	1.46	14.74	7.29	9.36	-0.20	0.05	0.18	0.30
*S*. *sp*. *nov*. 5	5	2	0.89	12.59	6.04	8.13	1.13	11.74	4.89	5.96	0.96	12.34	5.71	7.51	-0.21	0.07	0.24	0.36
*S*. *sp*. *nov*. 6	10	13	0.99	13.41	6.82	9.19	1.31	12.67	5.63	6.84	1.17	12.99	6.15	7.86	-0.24	0.06	0.21	0.34
*S*. *sp*. *nov*. 7	10	10	1.28	15.94	8.38	10.99	1.65	15.33	7.59	**8.89**	1.46	15.63	7.98	**9.94**	-0.22	0.04	0.10	0.24
*S*. *sp*. *nov*. 8	23	19	1.02	13.67	6.99	8.77	1.35	13.92	6.67	7.61	1.17	13.78	6.85	8.25	-0.24	-0.02	0.05	0.15

n, number of individuals considered; FW1, Femur I width; F3L, Femur III Length; TL, Thorax Length; TW, Thorax Width; ♀/♂, measurements of females over males’.

### Comparative analyses

To test whether climatic and elevation variables influenced male and female body size, we fitted Generalized Linear Mixed Models (GLMMs) using Markov Chain Monte Carlo algorithms, as implemented in the R [67] package ‘MCMCglmm’ [68]. The package makes use of the flexible and widely employed GLMMs whilst marginalizing the random effects in a robust manner (compared to other, currently available packages). For models including phylogenetic effects, such as ours, a vector containing a tree topology must be associated with the inverse relationship matrix A^-1^. This matrix is, in turn, formed by assigning the tree topology to the pedigree argument of MCMCglmm [68]. The method has been used in a variety of studies, such as to estimate the patterns of evolution in anuran vocal sexual signals [69] and to test Darwin’s naturalization hypothesis in plants [70]. We used Femur I Width, Femur III Length, and Thorax Length and Width as dependent variables, and Sex, Elevation, TS, MTWT, MTCT and MAP as independent variables, and a vector containing the species tree topology (which resulted from the *BEAST analysis) as a random variable. Note that the latter allowed us to account for the phylogenetic non-independence of species [71]. The models fitted a univariate normal response. The full, saturated models included the first order interaction between sex and all other independent variables, and we removed non-significant interaction terms by backward elimination. Models were run for 5,500,000 iterations after a burn-in of 1000 iterations and a thinning interval of 500 iterations. The proportion of the total variance in a given model was accounted for by the random variable tree topology, which was calculated for each model. We further ensured that Effective Sampling Sizes (ESS) were adequate (> 10000). The significance of the predictors was determined when the 95% credible intervals of the effect size excluded zero [e.g. 68]. Finally, for each model, we also determined the extent of the phylogenetic signal by calculating Pagel’sλ [72].

To test if the divergence of male body size has been greater than female body size in *Sphenarium* species (Rensch’s rule), we used the phylogenetic independent contrasts method [35], as implemented by the R package ‘caper’ [73], to control for the phylogenetic non-independence of species [71]. Since outliers can seriously affect the parameter estimates for any regression model we removed automatically outliers with studentized residuals > ±3 [74]. A key assumption of the contrasts method is that the standardized contrasts are independent from their estimated nodal values [35]. This assumption was verified by plotting the standardized contrasts against their estimated nodal values using the ‘plot’ function provided by ‘caper’. We then tested the allometric relationship between *log*(male) (dependent variable) and *log*(female) (independent variable) body size (Femur I Width, Femur III Length, Thorax Width and Length) by fitting four major axis regressions (model II regression, MA [75]) using the phylogenetic independent contrasts [76]. Rensch’s rule predicts the slope of male on female size to be significantly larger than 1. Since the mean value of contrasts is expected to be zero, the MA regression was forced through the origin [70]. We provided the slope of major axis regressions (*β*), as well as their 95% lower and upper confidence intervals, which were calculated using the R package ‘smatr’ [77,78].

## Results

### Genetic data

We successfully sequenced 1065 bp of CO1, 486 bp of CO2, 336 bp of 12S, 318 bp of H3 and 311 bp of ITS2. For some individuals, mostly outgroup taxa, we obtained shorter CO2, 12S and ITS2 sequences because indels were present. We could not obtain reliable sequences from all five loci for some individuals. In some cases, multiple copies (for ITS2) or pseudogenes (for H3 and CO2) were detected. The number of sequences per locus obtained was as follows: 76 for CO1, 54 for CO2, 58 for 12S, 49 for H3 and 41 for ITS2. The final dataset for the phylogenetic analysis comprised 82 terminals and 2,524 aligned nucleotides. In this dataset CO2 sequences showed the highest percentage of parsimony informative sites (39.8% of 1065 bp), followed by CO1 (32.7% of 486 bp), 12S (24% of 336 bp), H3 (10% of 318 bp) and ITS2 (8.2% of 311 bp).

### Definition of taxonomic units

**Fig 2:**
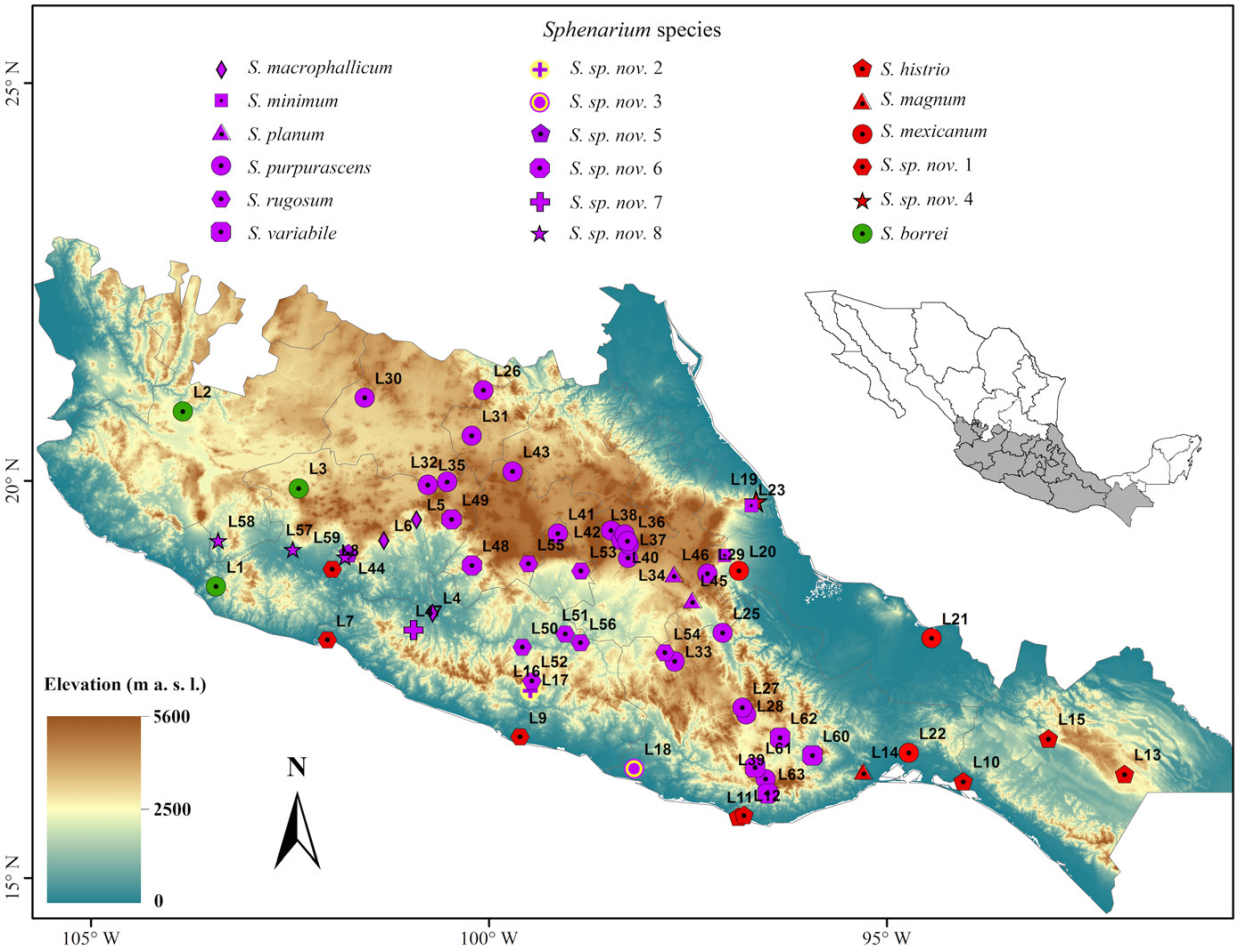
Sampling localities of *Sphenarium* (L1-L63) included in this study and geographic distribution of the 18 identified species within the genus. This map is based on the digital elevation model developed for Mexico, including only the Mexican states where the genus *Sphenarium* is distributed.

We identified a total of 18 morphologically and/or genetically distinct lineages within *Sphenarium* (Table 4). Eight lineages represented probably new species within the genus; other wise, they corresponded to previously recognized species, but in most cases they only comprised individuals from particular geographic provinces (Fig 2). Within the morphological pattern of differentiation, we identified eight species: *S*. *macrophallicum*, *S*. *variabile*, *S*. *mexicanum*, *S*. *histrio* (*S*. *m*. *histrio* localities in southern Mexico), *S*. *magnum* (previously synonymized within the intermediate form between *S*. *m*. *mexicanum* and *S*. *m*. *histrio*), *S*. *purpurascens* (*S*. *p*. *purpurascens* localities in central Mexico highlands), *S*. *rugosum* (*S*. *rugosum* localities in the eastern portion of Balsas River Basin), and *S*. *sp*. *nov*. *7* (*S*. *rugosum* in the southern middle portion of the Balsas River Basin). Within the cryptic genetic pattern of differentiation, we identified eight species, most of them probably representing new taxa: *S*. *planum* (*S*. *p*. *purpurascens* localities from the Tehuacan Valley, previously synonymized within the intermediate form between *S*. *p*. *purpurascens* and *S*. *p*. *minimum*), *S*. *sp*. *nov*. *1* (*S*. *m*. *histrio* localities in northern Pacific Costal Plains and western portion of the Balsas River Basin), *S*. *sp*. *nov*. *2* (*S*. *m*. *histrio* localities in the Sierra Madre del Sur of Guerrero), *S*. *sp*. *nov*. *3* (*S*. *m*. *histrio* found in the Pacific Costal Plain of Oaxaca), *S*. *sp*. *nov*. *4* (*S*. *m*. *histrio* localities in the northeastern portion the Mexican Volcanic Belt), *S*. *sp*. *nov*. *5* (*S*. *p*. *purpurascens* found in the western portion of the Balsas River Basin), *S*. *sp*. *nov*. *6* (*S*. *rugosum* localities found in north-eastern portion of the middle Balsas River Basin) and *S*. *sp*. *nov*. *8* (*S*. *rugosum* localities found in the northwestern portion of the Balsas River Basin). Finally, within the morphological and genetic pattern of differentiation we recognized *S*. *borrei* and *S*. *minimum* (*S*. *p*. *minimum* localities) as separate species.

**Table 4:** Identified *Sphenarium* species using the integrative taxonomy framework. The current taxonomic classification and localities included within each identified species are also shown.

Identified species	Pattern of differentiation	Current species ^A^	Localities
*S*. *borrei*	M & G	*S*. *borrei*	L1—L3
*S*. *macrophallicum*	M	*S*. *macrophallicum*	L4—L6
*S*. *histrio*	M	*S*. *m*. *histrio*	L10—L13, L15
*S*. *mexicanum*	M	*S*. *m*. *mexicanum*	L20—L22
*S*. *magnum*	M	*S*. *m*. *mexicanum* ^B^	L14
*S*. *minimum*	M & G	*S*. *p*. *minimum*	L23, L24
*S*. *purpurascens*	M	*S*. *p*. *purpurascens*	L25—L43
*S*. *planum*	G	*S*. *p*. *purpurascens* ^C^	L45, L46
*S*. *rugosum*	M	*S*. *rugosum*	L50—L56
*S*. *variabile*	M	*S*. *variabile*	L60—L63
*S*. *sp*. *nov*. *1*	G	*S*. *m*. *histrio*	L7—L9
*S*. *sp*. *nov*. *2*	G	*S*. *m*. *histrio*	L17, L16
*S*. *sp*. *nov*. *3*	G	*S*. *m*. *histrio*	L18
*S*. *sp*. *nov*. *4*	G	*S*. *m*. *histrio*	L19
*S*. *sp*. *nov*. *5*	G	*S*. *p*. *purpurascens*	L44
*S*. *sp*. *nov*. *6*	G	*S*. *rugosum*	L48, L49
*S*. *sp*. *nov*. *7*	M	*S*. *rugosum*	L47
*S*. *sp*. *nov*. *8*	G	*S*. *rugosum*	L57—L59

M, morphological differentiation; G, genetic differentiation; M & G, morphological and genetic differentiation.

^A^ taxonomic classification recognized by Eades [38] based on Boyle [36] and Kevan [37].

^*B*^ specimens corresponding to the intermediate form between *S*. *m*. *mexicanum* and *S*. *m*. *histrio*, within which *S*. *magnum* was synonymized.

^*C*^ specimens corresponding to the intermediate form between *S*. *p*. *purpurascens* and *S*. *p*. *minimum*, within which *S*. *planum* was synonymized.

### Phylogenetic relationships

The Bayesian analysis based on total evidence data completely resolved the higher-level phylogenetic relationships with most nodes well supported with posterior probability (*PP*) values higher than 0.94 (Fig 1). All included Pyrgomorphidae and species within the tribe Sphenariini (*P*. *scudderi*, *M*. *xiangchengensis*, *M*. *kingdoni*, *M*. *xizangensis* and *Y*. *coriacea*), with the exception of *J*. *oviedensis*, formed a monophyletic group, and a close phylogenetic relationship between most Sphenariini species and other American Pyrgomorphidae (*P*. *pueblensis*, *S*. *nobilis* and *S*. *crassicornis*) was identified. Despite the fact that our results indicated that the subtribe Sphenariina (comprising the genus *Sphenarium*, *Prosphena* and *Jaragua*) was paraphyletic, a sister relationship between the genus *Sphenarium* and *Prosphena*, as well as the monophyly of these genera were strongly supported (*PP* = 1).

**Fig 3:**
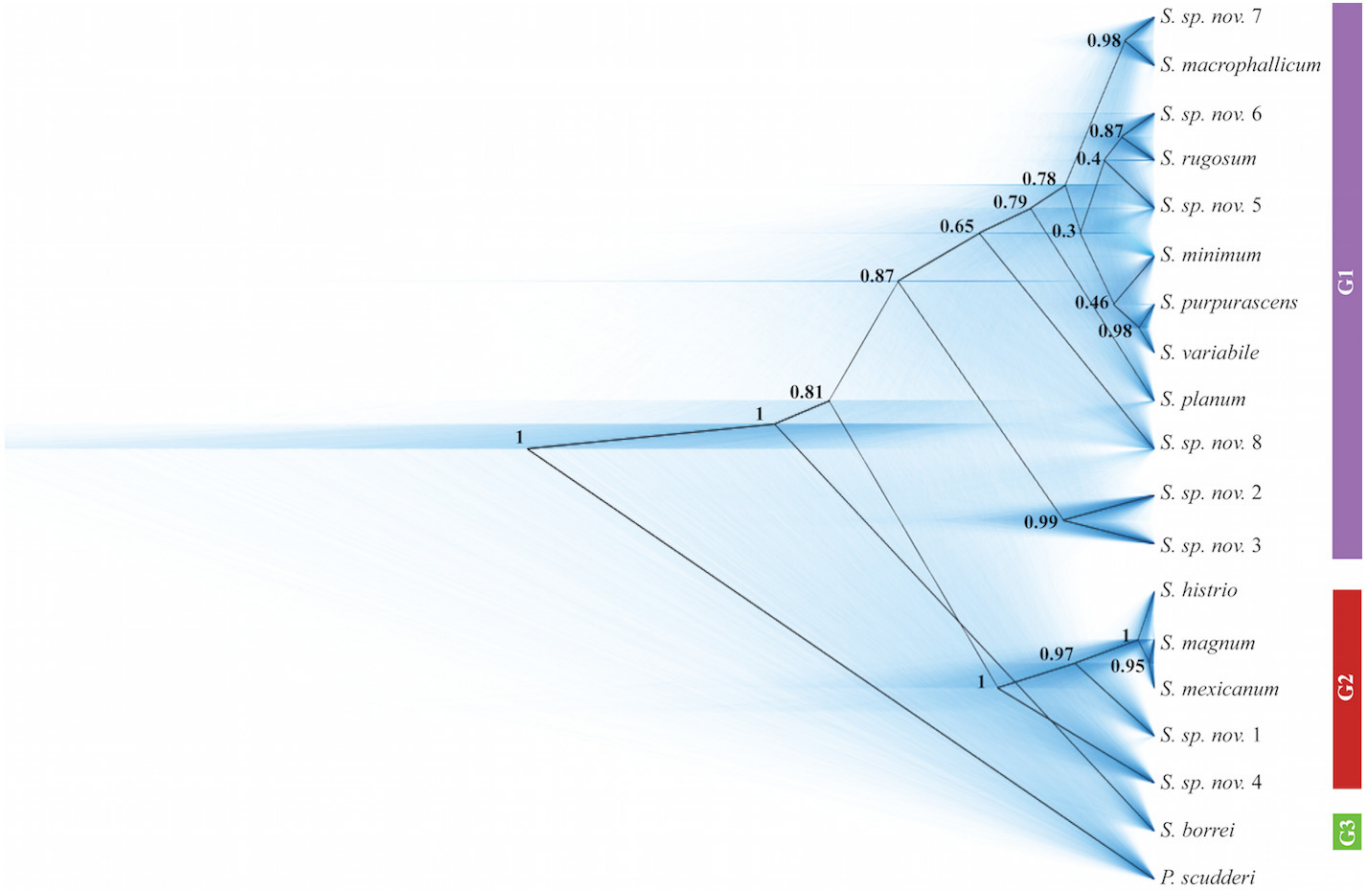
Consensus species tree of the 18 identified taxa of *Sphenarium*. The consensus species tree (in black) is embedded in all sampled trees (in blue) of the Markov Chain Monte Carlo chain of the species tree analysis including the total genetic evidence obtained from *Sphenarium* and *Prosphena* individuals. Higher tree densities represent high levels of certainty. Numbers before the nodes indicate posterior probabilities values. G1, Monophyletic Group 1; G2, Monophyletic Group 2; G3, Monophyletic Group 3.

Within the genus *Sphenarium*, we identified three major geographically structured monophyletic groups in the Concatenated Analysis (CA) and Species Tree Analysis (STA) (Fig 3). The Group 1 [*PP* = 0.92 (CA) and 0.87 (STA)] comprised species distributed in the inner basins and highlands of central Mexico (*S*. *machiphallicum*, *S*. *minimum*, *S*. *planum*, *S*. *purpurascens*, *S*. *rugosum*, *S*. *variabile*, *S*. *sp*. *nov*. *2*, *S*. *sp*. *nov*. *3 S*. *sp*. *nov*. *5*, *S*. *sp*. *nov*. *6*, *S*. *sp*. *nov*. *7* and *S*. *sp*. *nov*. *8*). The Group 2 [*PP* = 1 (CA and STA)] included the species distributed in the costal lowlands of central and southern Mexico, and Sierra Madre de Chiapas (*S*. *histrio*, *S*. *magnum*, *S*. *mexicanum*, *S*. *sp*. *nov*. *1* and *S*. *sp*. *nov*. *4*). The Group 3 [*PP* = 1 (CA and STA)] comprised solely *S*. *borrei* lineages restricted to northwestern portions of the Mexican Volcanic Belt (see Fig 2 for species distribution and Fig 3 for phylogenetic relationships).

In both analyses we also recovered a close phylogenetic relationship between Group 1 and Group 2 [*PP* = 0.53 (CA) and 0.81 (STA)], and *S*. *borrei* lineages (Group 3) as the basal species in the genus. Moreover, within Group 1 the species *S*. *machiphallicum*, *S*. *minimum*, *S*. *purpurascens*, *S*. *rugosum*, *S*. *variabile*, *S*. *sp*. *nov*. *5*, *S*. *sp*. *nov*. *6* and *S*. *sp*. *nov*. *7* formed a monophyletic group [*PP* = 0.67 (CA) and 0.78 (STA)], which was closely related to *S*. *planum* [*PP* = 1 (CA) and 0.79 (STA)]. In both analyses, we also observed a strong (*PP* > 0.95) sister relationship among three pairs of species comprising *S*. *purpurascens*–*S*. *variabile*, *S*. *machiphallicum*–*S*. *sp*. *nov*. *7*, *S*. *sp*. *nov*. *2* –*S*. *sp*. *nov*. *3*, and *S*. *mexicanum*–*S*. *magnum*. In both analyses, phylogenetic relationships were similar and strongly supported (PP > 0.95) among species within Group 2. Within this group *S*. *sp*. *nov*. *4* was placed in the basal position, *S*. *sp*. *nov*. *1* was closely related to *S*. *histrio*, *S*. *mexicanum* and *S*. *magnum*.

The main difference between the two analyses performed was the placement of the species pair *S*. *sp*. *nov*. *2* –*S*. *sp*. *nov*. *3*. In the CA the species *S*. *sp*. *nov*. *2* –*S*. *sp*. *nov*. *3* was more closely related to the species group comprising *S*. *machiphallicum*, *S*. *minimum*, *S*. *planum*, *S*. *purpurascens*, *S*. *rugosum*, *S*. *variabile*, *S*. *sp*. *nov*. *5*, *S*. *sp*. *nov*. *6* and *S*. *sp*. *nov*. *7* (*PP* = 1); whereas in the STA the same species pair was placed in the basal position within Group 1. In addition, different relationships with *PP* values lower than 0.67 were obtained between the species and species pairs within the group comprising *S*. *machiphallicum*, *S*. *minimum*, *S*. *planum*, *S*. *purpurascens*, *S*. *rugosum*, *S*. *variabile*, *S*. *sp*. *nov*. *5*, *S*. *sp*. *nov*. *6* and *S*. *sp*. *nov*. *7* in both analyses.

### Morphological analyses

A total of 5441 *Sphenarium* grasshoppers (2508 females and 2933 males) were measured from the 63 sampled localities. The number of individuals considered within each species and sex ranged from 2 to 2204 (Table 3). *S*. *purpurascens* and *S*. *rugosum* were the species with the largest samples size (> 300 individuals each sex), whereas *S*. *sp*. *nov*. *5* had the lowest sample size (5 females and 2 males). Females and males showed considerable variation among the 18 taxa in all traits measured. In females, mean Femur I Width ranged from 0.85 to 1.38 mm, the Femur III Length ranged from 11.82 to 18.56 mm, the Thorax Length ranged from 5.9 to 9 mm, and the Thorax Width ranged from 7.94 to 11.42 mm. In males, mean Femur I Width ranged from 1.11 to 1.76 mm, the Femur III Length ranged from 10.69 to 16.73 mm, the Thorax Length ranged from 4.74 to 7.59 mm, and the Thorax Width ranged from 5.77 to 8.89 mm. For the measured traits, the largest species (*S*. *magnum*, *S*. *mexicanum*, *S*. *sp*. *nov*. *1* and *S*. *sp*. *nov*. *7*) were nearly 1.38 to 1.63-fold larger than the smallest ones (*S*. *minimum*, *S*. *planum*, *S*. *purpurascens*, *S*. *variabile* and *S*. *sp*. *nov*. *5*; Table 3).

In all species the Femur I was wider in males than in females, whereas the Thorax Length and Width was larger in females than males. The Length of the Femur III was larger in females than in males, except for S. *purpurascens* and *S*. *sp*. *nov*. *8*, which showed the inverse pattern (Table 3). The magnitude of sexual dimorphism varied notably among species and traits. For instance, *S*. *purpurascens*, showed the highest levels of sexual dimorphism in Femur I Width, whereas *S*. *planum* and *S*. *minimum* showed the highest values of sexual dimorphism in the other three traits. The magnitude of sexual dimorphism in the Femur I Width ranged from -0.14 to -0.35. For the Femur III Length varied from -0.04 to 0.15, in the Thorax Length ranged from 0.05 to 0.40, and in the Thorax Width ranged from 0.15 to 0.55 (Table 3).

### Altitude, climatic variation and body size

**Fig 4:**
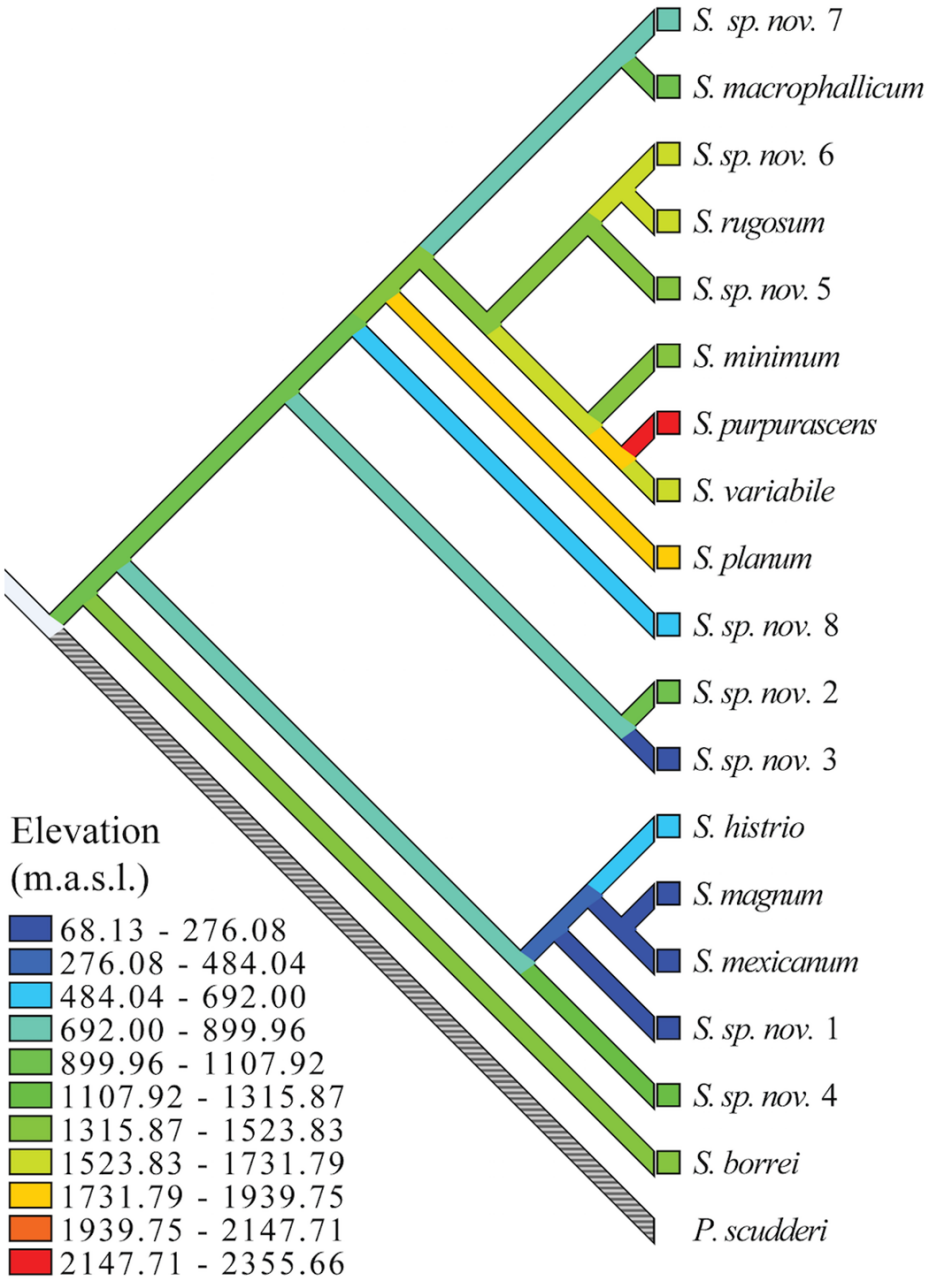
Parsimony ancestral reconstruction of the altitudinal distribution of the *Sphenarium* species performed in MESQUITE v. 3.0.2 [79]. For this analysis we used the species tree analysis topology and the mean elevation values estimated for each species.

After controlling for phylogenetic non-independence among *Sphenarium* species, the results of the MCMCglmm analysis indicated significant differences between females and males for the four morphological traits (Table 5). We found a positive and significant relationship between temperature during winter (MTWT) and Femur III Length and Thorax Width. In addition, temperature seasonality was positively related to the four traits. The elevation, Mean Temperature of the Wettest Trimester and Mean Annual Precipitation had no significant effect on body size (Table 5). The interactions between sex and all other independent variables were not significant (data not shown in simplified models), indicating a similar body size response to elevation and climatic variables between sexes. All models showed high λ values (λ > 0.94), indicating a strong phylogenetic effect on the relationships between the ecological and morphological variables. A character reconstruction of the altitudinal distribution of the species (Fig 4) indicated a mid elevation origin of the genus *Sphenarium*, considering that the inferred common ancestor and most species within the genus occupied intermediated elevations (around 800–1500 m), whereas low (<800 m) and highlands (>1500 m) distributions have been occupied independently several times in the genus.

**Table 5:** MCMCglmm models for the body size indicators and independent variables (sex, elevation and climatic parameters) of *Sphenarium* species. Significant interactions are denoted in bold numbers.

Morphologic trait	Source	Posterior Mean	Lower CI	Upper CI	ESS	*PP*
**FW1**, λ = 0.99 (0.98–1)						
	Intercept	1.48	-3.75	6.51	11308	0.56
	Sex	-0.25	-0.29	-0.20	10998	**0.00**
	Elevation	-0.16	-0.40	0.10	10998	0.21
	TS	1.22	0.23	2.16	10998	**0.02**
	MTWT	-5.50	-13.35	2.01	10998	0.14
	MTCT	4.87	-0.04	10.10	10998	0.06
	MAP	0.00	-0.31	0.33	11325	0.99
**F3L**, λ = 0.98 (0.88–1)						
	Intercept	3.23	-0.44	7.26	11507	0.08
	Sex	0.06	0.02	0.10	10998	**0.01**
	Elevation	-0.16	-0.34	0.04	10998	0.10
	TS	1.07	0.30	1.80	11474	**0.01**
	MTWT	-4.71	-10.25	1.45	11546	0.10
	MTCT	4.21	0.20	7.98	11421	**0.04**
	MAP	0.03	-0.21	0.30	10998	0.81
**TXL**, λ = 0.95 (0.70–1)						
	Intercept	0.57	-3.78	4.93	10998	0.79
	Sex	0.17	0.12	0.22	10998	**0.00**
	Elevation	-0.04	-0.27	0.18	10998	0.68
	TS	1.19	0.31	2.08	10998	**0.01**
	MTWT	-3.64	-10.37	2.91	10998	0.25
	MTCT	4.14	-0.33	8.60	10998	0.07
	MAP	0.03	-0.27	0.34	10998	0.84
**TXW**, λ = 0.94 (0.68–1)						
	Intercept	1.65	-2.25	6.10	10998	0.41
	Sex	0.28	0.23	0.34	10998	**0.00**
	Elevation	-0.05	-0.27	0.17	10998	0.61
	TS	1.19	0.30	2.04	10998	**0.01**
	MTWT	-4.56	-11.28	1.83	10998	0.14
	MTCT	4.49	0.23	9.10	10998	**0.03**
	MAP	-0.04	-0.33	0.27	11100	0.80

CI, 95% confidence interval; ESS, Effective Sampling Size; *PP*, Posterior probabilities values of Markov chain Monte Carlo analysis; FW1, Femur I Width; F3L, Femur 3 Length; TL, Thorax Length; TW, Thorax Width; TS, Temperature Seasonality; MTWT, Mean Temperature of The Wettest Trimester; MTCT, Mean Temperature of The Coldest Trimester; MAP, Mean Annual Precipitation; λ, Phylogenetic Signal of the model with lower and upper confidence interval values within parenthesis.

### Rensch’s rule

**Fig 5:**
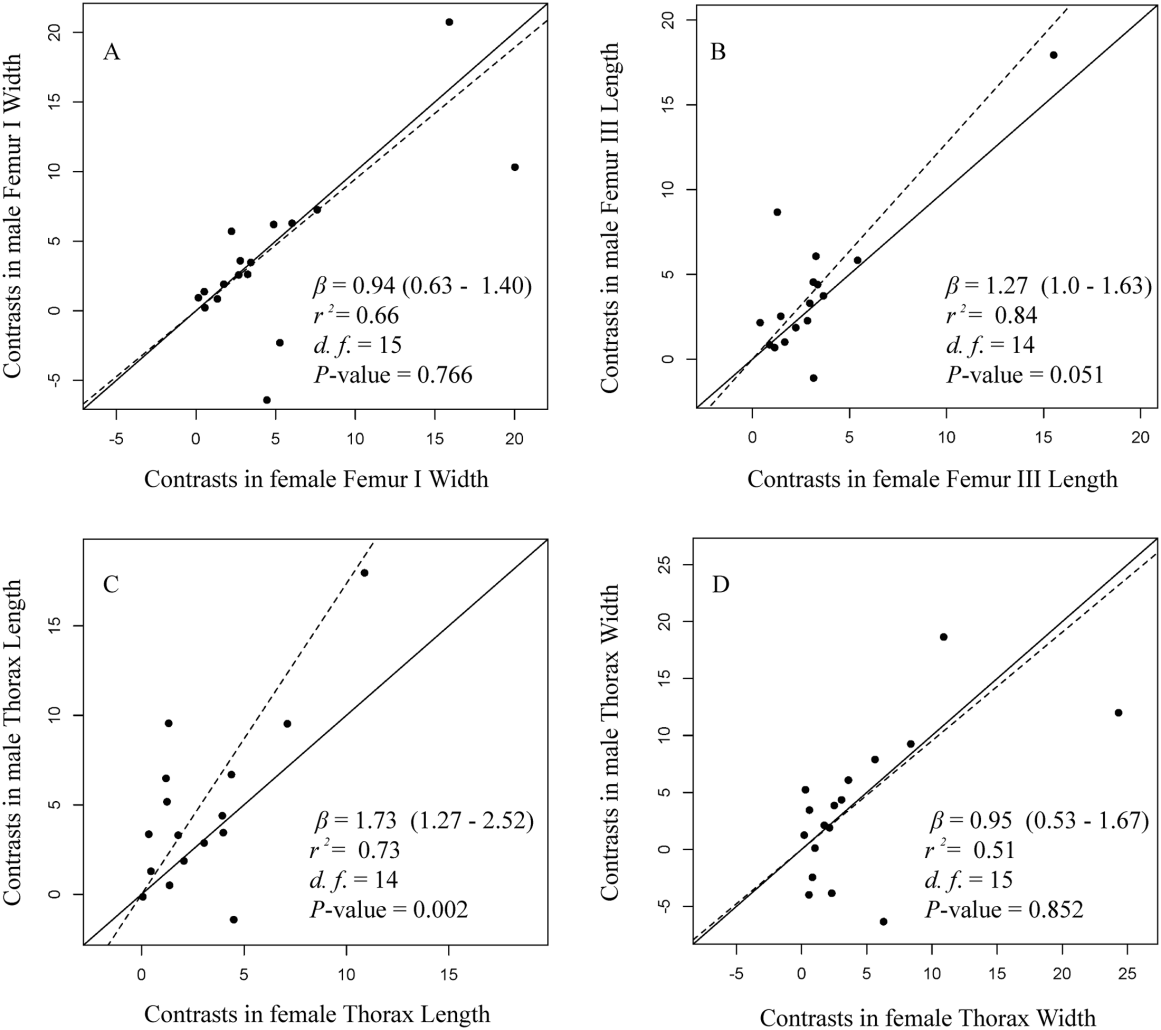
Model II major axis regressions of independent contrasts of body size indicators of males and females of *Sphenarium* species. The *P*-value from the comparison of each calculated slope *vs* slope = 1 (H_O_: slope not different from 1). *β*, slope with lower and upper confidence intervals at 95% probability within parenthesis; *r* ^*2*^, explained variance of the model; *d*.*f*., degrees of freedom.

**Fig 6:**
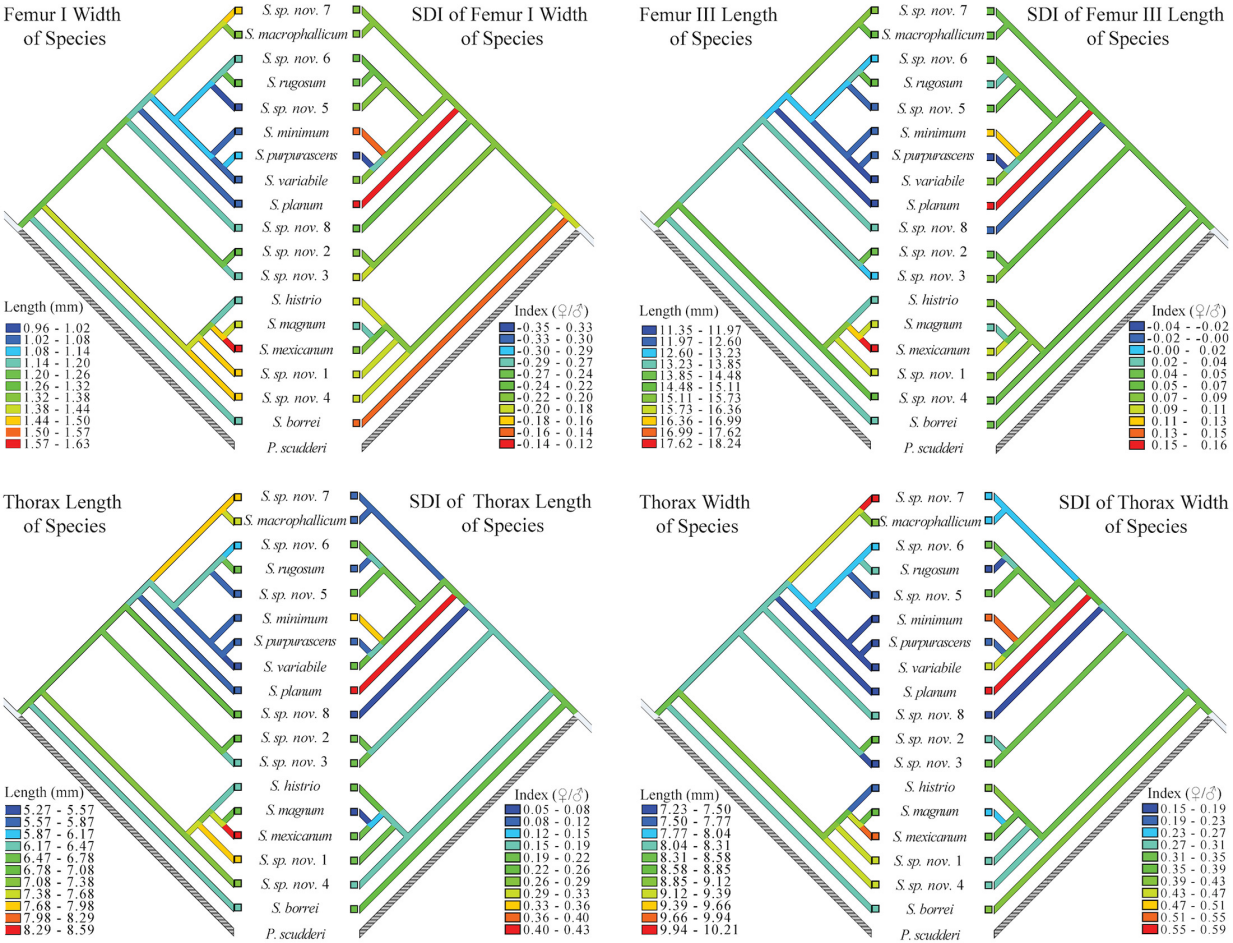
Parsimony ancestral reconstruction of the body size and the magnitude of SSD of the *Sphenarium* species performed in MESQUITE v. 3.0.2 [79]. For this analysis we used the species tree analysis topology and the mean values of SDI estimated for each species (considering females over males) for each trait.

The results of model II regressions of independent contrasts analysis indicated strong coevolution between females and males (Fig 5). Regressions of males’ over females’ traits resulted in slopes greater than 1.0 for Femur III and Thorax Lengths. However, only Thorax Length significantly differed from isometry (*β* = 1.0). In addition, in order to explore the evolutionary trends on body size and SSD we performed an ancestral character reconstruction using the mean species values and SDI from each trait. All traits measured resulted in similar pattern (Fig 6). Large body size and high levels of SSD have evolved independently several times throughout the evolutionary history of the group. Small body size and high levels of SSD occurred more frequently in species within Group 1 (Fig 6).

## Discussion

*Sphenarium* grasshoppers show considerable divergence in size during the diversification of the genus. Despite the fact that phylogenetic relationships are heavily affecting body size and climatic niche of *Sphenarium* species, we find (according to our hypotheses) that large *Sphenarium* species are associated with high temperatures during the winter. However, they are also associated with highly seasonal environments. Body size is not significantly related to precipitation or temperatures during the rainy season. In addition, *Sphenarium* females and males respond similarly to the climatic differences, and the evolutionary divergence in the thorax length has been greater in males than in females.

Our results suggest that during benign winters, the window for development and reproduction may increase, allowing grasshoppers to achieve larger body sizes. Conversely, when mean temperatures are lower, body sizes become smaller. Similar body size clines associated with decreasing temperatures have been observed in other insects at higher latitudes [7–10]. Smaller body sizes at low temperatures are commonly explained by natural selection favouring faster development by decreasing development time (reducing the number of nymphal instars or diapause [7] or increasing growth rates [33]).

The climatic body size cline of *Sphenarium* grasshoppers probably reflects their life history adaptability. The nymphs of *Sphenarium* emerge mainly in the beginning of the rainy season and adults die in the winter. However, there is considerable variation in emergence times and life cycle lengths at inter- and intraspecific levels. In *S*. *purpurascens*, the taxa with the highest altitudinal distribution, hatching occurs in the middle of June in central Mexico (<2200 m.a.s.l.). The first organisms reach sexual maturity at the end of August, the peak of the reproductive season occurs by middle October, and they die off when temperatures drop drastically at the beginning of December [46]. On the other hand, in *S*. *sp*. *nov*. *1* at lowlands of the western portion of the Balsas River Basin (239 m.a.s.l) the peak of the reproductive season occurs in middle September, whereas in *S*. *sp*. *nov*. *8* few adults and mostly last instar nymphs at higher altitudes (1074 m.a.s.l.) can be found at that time (Sanabria-Urban *pers*. *obs*.).

In general, smaller body sizes are favored in seasonal environment because both maturation time and body size are constrained by weather and food availability [8,80]. However, contrary to our initial expectation, our results indicate that larger grasshopper species are associated, on average, with a greater variability on temperature (i.e. high seasonality). The pattern found in *Sphenarium* could differ from those found in other taxa mainly because species with a wider spatial and altitudinal distribution can be exposed to more heterogeneous environments (which accounted for greater variation in seasonality) than species with a narrow distribution. Moreover, species with a narrow distribution can be exposed to low or high temperature seasonality (see Table 2). Thus, as result of the high levels of environmental variation, high levels of phenotypic variation would be expected too. In order to test this possibility, we correlated the coefficients of variation of phenotypic traits of *Sphenarium* species on their estimated temperature seasonality. In general high levels of phenotypic variation were positively related with high temperature seasonality (S3 Table).

The extensive interspecific variation in morphological traits could suggest local adaptation, which could be the result of adaptive genetic variation, and/or phenotypic plasticity. Biotic and abiotic factors like temperature and food availability can affect growth and development times. For instance, insects grown under high temperatures and high quality and/or quantity of food resources can reach large body sizes [81,82] and females and males can respond in different ways to the environment [26]. However, in *Sphenarium* females and males respond similarly to the environmental differences.

In Mexico, western and eastern mountain ranges generate large environmental heterogeneity. Temperate, dry and rain forest and even desserts occur in short distance apart at the same latitude. This variation in plant communities results in differential availability of both, food quantity and quality. Because *Sphenarium* are generalist herbivores, they can feed on a wide variety of plants across their altitudinal range. Their diet includes seasonal species that produce leaves and flowers during the growing season of the grasshoppers (e.g. *Dahlia coccinea*, *Verbesina virgate*, *Datura stramonium*, *Tithonia sp*.), perennial species (e.g. *Montanoa tomentosa*, *Eupatorium petiolare*, *Budleia cordata*) and even crop plant and trees, which provide a continuum of food supply during development and reproductive season of grasshoppers [83,84]. The potential continuum of food supply during the life cycle of the grasshoppers may reduce the indirect relationship between precipitation and size that has been found in other grasshoppers [34], and may explain why we did not find the significant relationship between body size and precipitation that has been found in other ectotherm species [62,85].

Decreasing the time to maturity at low temperatures may have negative effects on the fitness of individuals by reducing reproductive success via small body sizes [10,12,86]. The smallest species of *Sphenarium* (*S*. *purpurascens*, *S*. *minimum*, *S*. *planum*, *S*. *variabile and S*. *sp*. *nov*. *5*) probably have lower fecundity than larger species, but they have been able to colonize highlands. However, at intraspecific level in *S*. *purpurascens* this trade-off between maturation time and body size does not seem to exist. Early maturation and large body size are associated with high mating success in both sexes [44,45].

The divergence in body size among *Sphenarium* species can be explained by natural selection operating on their life cycles, but also by sexual selection. The relationship between male and female thorax length was significantly hyperallometric, indicating a greater evolutionary divergence in male body size, even though the other traits showed isometric relationships. Hyperallometry is typically explained by strong sexual selection acting on male body size [21,23,25,87]. According to the Rensch’s rule, because thorax length is larger in females than males, the greater divergence on males may explain why there is a low SSD in thorax length of larger species (Fig 6). In *S*. *purpurascens* body size, including thorax length increase mating success in both, females and males [44,45], even thought the impact of sexual selection on males can be stronger than in females. On the other hand, isometry in Femurs I and III and thorax width can be explained by genetic [88], phylogenetic [89], developmental [90], and/or physiological [91] constraints, differences in the magnitude and/or direction of selection between species or even that size is not under any evolutionary pressure.

SSD is the result of differential selection among sexes, and the balance between natural selection and sexual selection in a given species [16,92]. In any case, the different patterns associated with the evolution of body size and SSD in *Sphenarium* (see Fig 6) may suggest that in some species natural selection has been stronger than sexual selection. Natural selection on developing time associated with a short reproductive season can favour small body sizes. However, positive directional sexual selection on thorax length could explain a greater divergence in males than in females. Small body sizes and high SSD levels on thorax length (e. g. *S*. *planum* and *S*. *minimum*) can be explained by strong natural selection associated with a short reproductive season, and low levels of sexual selection on male body size. On the other hand, large body sizes and low SSD levels (e. g. *S*. *magnum* and *S*. *mexicanum*) can be expected under strong sexual selection on males and lower levels of natural selection associated with a long growing and reproductive season, which also favors large body sizes in both females and males. Moreover, small body sizes and low SSD (e. g. *S*. *purpurascens* and *S*. *variabile*) could result from strong sexual and natural selection on body size and maturation time in places with short reproductive seasons [44,45].

In *S*. *purpurascens* body size and maturation times are under strong sexual and natural selection in both males and females. Individuals maturing earlier and with larger body sizes usually attain high mating success [45,46]. However, pre- and post-copulatory sexual selection could be stronger on males than on females. Female and males can mate multiple times, and sperm competition is very likely [93,94]. After copulation, large males remain in a post-insemination association with their mates. A male can spend as many as 17 days mounted on a female, and guarding duration is related to both male and female body sizes [93].

The diversification of the genus *Sphenarium* could be associated with Quaternary climatic fluctuations, which probably cause the vicariance of ancestral populations throughout recurrent shifts in their altitudinal and spatial distribution. The low mobility of these univoltine and flightless grasshoppers, plus the combination of strong natural and sexual selection on adult body size and maturation times could enhance the genetic isolation and consequently the speciation of these Neotropical grasshoppers. Genetic differentiation, but also high levels of phenotypic plasticity could explain the diversification of the clade. We are currently conducting the taxonomic revision of the genus and evaluating the genetic variance and plasticity levels in body size and maturation time within and between species. Perhaps widely distributed and more variable species will show the highest levels of phenotypic plasticity.

## Supporting Information

S1 Table Sampling localities, specimens and GenBank accession numbers of the *Sphenarium* individuals and outgroup species included in this study. ID, locality identifier; VN, voucher number. ^A^ species identification based on Boyle [36] and Kevan [37,95] and by comparing our samples with museum specimens. ^*B*^ Intermediate form between *S*. *m*. *mexicanum* and *S*. *m*. *histrio*. ^*C*^ Intermediate form between *S*. *p*. *purpurascens* and *S*. *p*. *minimum*.
(XLSX)

S2 Table *P-distance* values between *CO1* sequences of the identified *Sphenarium* species. *P-distance* percentages were estimated using pairwise comparisons of evolutionary divergence implemented in MEGA v. 6.0.6 [53]. Standard errors were base on a 500 replicates bootstrap analysis. Bold numbers indicate *P-distance* values lower than 2%.
(XLSX)

S3 Table Coefficients of variation associated to the mean values of species of the morphologic traits and temperature seasonality. ^A^ Correlation analysis between coefficients of variation of Temperature Seasonality and all morphologic traits in both females and males, of *Sphenarium* species (*d*.*f*. 16). Significant correlations are denoted in bold. CV, coefficient of variation; FW1, Femur I Width; F3L, Femur 3 Length; TL, Thorax Length; TW, Thorax Width; TS, Temperature Seasonality; *r*, correlation values; *P*-values, significance values.
(XLSX)
