# Correction: Body Size Adaptations to Altitudinal Climatic Variation in Neotropical Grasshoppers of the Genus *Sphenarium* (Orthoptera: Pyrgomorphidae)

**DOI:** 10.1371/journal.pone.0148329

**Published:** 2016-02-03

**Authors:** The PLOS ONE Staff

Table 1 appears incorrectly in the published article. Please see the corrected Table 1 below.

**Table 1:** List of amplified loci indicating their approximate size (bp), annealing temperature (Tm) and the pairs of primers used for the PCR reactions.

Loci	Size (bp)	Tm (°C)	Primer ^A^	Sequence (5'-3')
F1	3600	50	ORMET [48]	CATAAGCTAATGGGTTCATAC
			ORRLYS [48]	GAGACCAGTACTTGCTTTCAGTCATC
F2	2100	50	OR16SN ^B^	AGAAACCGACCTGGCTCACGC CGG
			OR12SN ^B^	CGTGCCAGCAGCCGCGGTTATACG
CO1	1180	58	SPHCO1F ^C^	TAGATCATCAATGGTTAATACAGG
			SPHCO1R ^C^	CTGATATGAGTGTTCTGCAGGAGG
CO2	550	58	C2J3138 [49]	GGAGCTTCACCATTAATAGAACA
			C2N3661 [49]	CCACAAATTTCTGAACATTGACCA
12S	360	58	SRJ14233 [49]	AAGAGCGACGGGCGATGTGT
			SRN14588 [49]	AAACTAGGATTAGATACCCTATTAT
H3	329	60	HexAF [50]	ATGGCTCGTACCAAGCAGACGGC
			HexAR [50]	ATATCCTTGGGCATGATGGTGAC
ITS2	320	60	CAS5p8sFc [51]	TGAACATCGACATTTYGAACGCACAT
			CAS28sB1d [51]	TTCTTTTCCTCCSCTTAYTRATATGCTTAA

^A^ source of primers is indicated within brackets and superscript letters.

^B^ designed by H. Song

^C^ designed by S. Sanabria-Urbán

The legend of Fig 1 is incomplete in the published article. Please see Fig 1 and its complete legend below.

**Fig 1:**
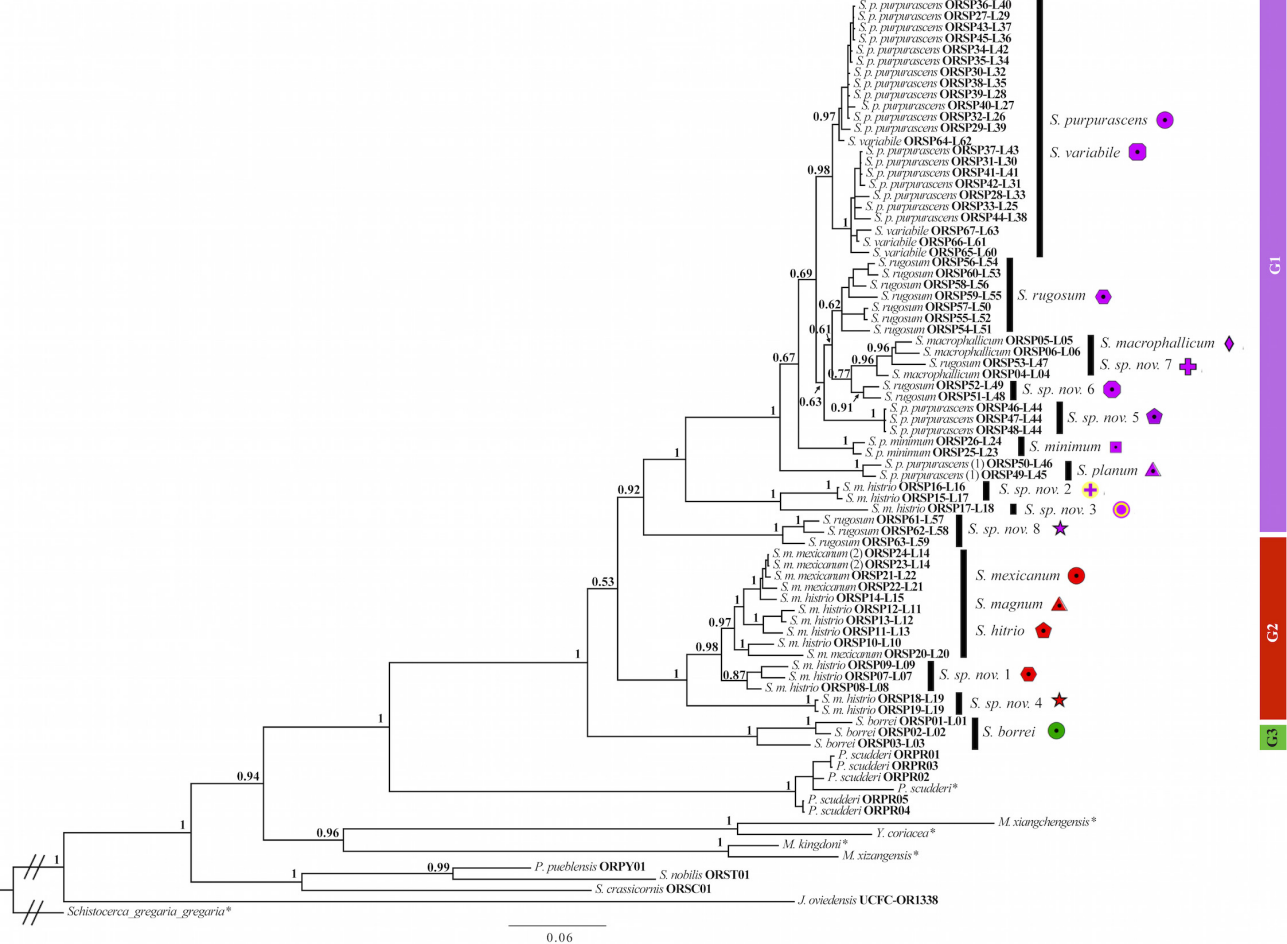
Phylogeny based on a concatenated Bayesian analysis of the total genetic evidence retrieved from 67 *Sphenarium* and 15 outgroup taxa. Tip labels indicate current taxonomic classification, voucher numbers and locality ID for all included terminals, except for those whose genetic information was retrieved from GenBank (*). Black vertical bars indicate the phylogenetic position of the identified species based on our integrative taxonomy approach (names and coloured symbols in front of the black bars). Particularly, the black vertical bars indicating the phylogenetic position of *S*. *purpurascens*, *S*. *variabile*, *S*. *macrophallicum*, *S*. *sp*. *nov*. *7*, *S*. *mexicanum*, *S*. *magnum* and *S*. *histrio* represent cases where species differentiation was primarily morphological and they did not separate in individual monophyletic groups (See methods and results sections for details). The numbers positioned closely to the nodes indicate posterior probability values. G1, Monophyletic Group 1; G2, Monophyletic Group 2; G3, Monophyletic Group 3. *S*. *p*. *purpurascens* (1) intermediate form between *S*. *p*. *purpurascens* and *S*. *p*. *minimum*. *S*. *m*. *mexicanum* (2) intermediate form between *S*. *m*. *mexicanum* and *S*. *m*. *histrio*.

The fourth paragraph of the Methods section under the subheading “Phylogenetic reconstruction” is erroneously included in the published article, and should be omitted.
